# A dietary combination of red yeast rice, phytosterol ester and lycopene ameliorates hypercholesterolemia by regulating gut microbiota and activating hepatic FXR-LDLR/ABCG pathway in mice

**DOI:** 10.3389/fmicb.2025.1622818

**Published:** 2025-08-07

**Authors:** Jingxian Xu, Xin Huang, Fei Pei, Yuzhu Chen, Cunzheng Zhang, Lingling Zhao, Hua Zhang, Jindong Zhang, Liping Duan

**Affiliations:** ^1^Department of Gastroenterology, Peking University Third Hospital, Beijing, China; ^2^Beijing Key Laboratory for Helicobacter pylori Infection and Upper Gastrointestinal Diseases, Beijing, China; ^3^Department of Pathology, Peking University Third Hospital, Peking University School of Basic Medical Sciences, Peking University Health Science Center, Beijing, China; ^4^H&H Group, H&H Research, China Research and Innovation Center, Guangzhou, China

**Keywords:** red yeast rice, hypercholesterolemia, gut microbiota, bile acid metabolism, FXR-LDLR/ABCG5/8 pathway

## Abstract

**Background:**

Excessive nutrition intake is a well-established contributor to obesity and hypercholesterolemia, both of which pose substantial risks to cardiovascular health. Statins, which are widely prescribed for managing serum cholesterol levels, are sometimes discontinued owing to adverse reactions. In contrast, dietary components have shown promise in lowing lipid lowering potential with a relatively higher safety profile, although the underlying mechanisms remains incompletely understood.

**Objectives:**

This study aimed to investigate the role and underlying mechanism of a dietary combination comprising red yeast rice (RYR), phytosterol ester, and lycopene (RPL), in mitigating hypercholesterolemia.

**Methods:**

High-fat, high-cholesterol (HFHC)-fed C57BL/6J mice were administered either the RPL combination (low and high dose) or simvastatin. The effects of these interventions on obesity, serum cholesterol, and glucose tolerance were evaluated. Mechanistic insights were gained through fecal 16S rRNA sequencing, targeted metabolomic profiling, and molecular analysis of liver and intestinal tissues using western blotting, qPCR, and immunofluorescence techniques.

**Results:**

Compared to the HFHC group, low and high doses of the RPL combination reduced serum low-density lipoprotein cholesterol (LDL-C) levels by 33 and 20%, respectively, whereas simvastatin achieved a 22% reduction. Both doses of RPL significantly lowered serum total cholesterol (TC) levels and alleviated obesity in mice, effects not observed with simvastatin. Mechanistically, the RPL combination reshaped the gut microbiota, specifically increasing the abundance of *Bifidobacterium* and decreasing that of *Clostridium*, *Ruminococcus* and *Eubacterium*. Additionally, the RPL combination modulated bile acids profiles, leading to an increased proportion of hyodeoxycholic acid (HDCA) and a decreased level of omega-muricholic acid (ω-MCA). Furthermore, the altered gut microbiota and ω-MCA levels activated the hepatic FXR-LDLR/ABCG5/8 pathway, promoting cholesterol excretion into feces and thereby alleviating hypercholesterolemia. The increased proportion of HDCA suppressed lipid absorption, further facilitating its excretion in feces.

**Conclusion:**

The dietary combination of RPL effectively lowers serum cholesterol by regulating gut microbiota, influencing bile acid metabolism, and enhancing cholesterol excretion. This study offers a novel and promising strategy for the clinical management of hypercholesterolemia.

## Introduction

The global prevalence of obesity and hypercholesterolemia has reached alarming proportions, primarily attributable to excessive caloric intake and increasingly sedentary lifestyles. These conditions are major risk factors for cardiovascular diseases (CVDs), which continue to be the leading cause of mortality worldwide. Statins, the cornerstone of cholesterol-lowering therapy, function by inhibiting 3-hydroxy-3-methylglutaryl-coenzyme A (HMG-CoA) reductase, a pivotal enzyme in cholesterol biosynthesis. Despite their efficacy, statins are associated with adverse effects such as myopathy, liver dysfunction, and gastrointestinal disturbances, leading to discontinuation in a significant proportion of patients (Ward et al., 2019; Newman, 2022).

It has been reported that various dietary components possess cholesterol-lowering properties and can serve as adjuncts for serum lipid management (Makhmudova et al., 2021; Banach et al., 2022; Belorio and Gómez, 2022; Khalid et al., 2023). Red yeast rice (RYR), a product of fermented japonica rice by Monascus (Gong et al., 2024), has a storied history spanning over two millennia and is commonly incorporated into daily foods such as fermented bean curd and red velvet cake. RYR is frequently combined with substances operating through different mechanisms to ameliorate hyperlipidemia, metabolic-associated fatty liver disease (MAFLD), and atherosclerosis (AS) (Cicero and Colletti, 2016; Cicero et al., 2017; Derosa et al., 2018; Marazzi et al., 2019). Phytosterol, abundant in vegetable oils and grains, competitively reduces intestinal cholesterol absorption (Babawale et al., 2018). By undergoing esterification with fatty acids, phytosterol can be converted into phytosterol ester, thereby enhancing its solubility. Given that phytosterol may interfere with the absorption of carotenoids such as lycopene (Gao et al., 2023), we primarily combined RYR and phytosterol ester, supplemented with additional lycopene. Although dietary supplements containing RYR have demonstrated promise in reducing serum cholesterol levels with fewer side effects compared to synthetic statins, the precise mechanisms underlying RYR’s lipid-lowering effects remain poorly understood, particularly its impact on gut microbiota and bile acid metabolism. Our objective is to provide preclinical evidence supporting the potential application of this combination in human populations.

The gut microbiota plays a pivotal role in regulating host metabolism, including cholesterol homeostasis. Recent studies have underscored the intricate interplay between gut microbiota, bile acid metabolism, and cholesterol excretion. Bile acids, synthesized from cholesterol in the liver, are modified by gut bacteria and serve as signaling molecules that influence lipid and glucose metabolism (Luo et al., 2020). The farnesoid X receptor (FXR), a nuclear receptor activated by bile acids, regulates the expression of genes involved in cholesterol transport, such as the low-density lipoprotein receptor (LDLR) and ATP-binding cassette transporters G5/G8 (ABCG5/8). These pathways are crucial for maintaining cholesterol balance and preventing hypercholesterolemia (Vourakis et al., 2021; Cai et al., 2022; Xu et al., 2023). After absorption in the small intestine, dietary cholesterol is taken up by the liver and transported into the bloodstream with the assistance of apolipoproteins (Luo et al., 2020). Excess cholesterol can be excreted from the body via reverse transport pathways, in which cholesterol transporters such as LDLR, SR-BI, and ABCG5/8 are closely involved. It has been reported that the microbiota-gut-liver axis constitutes an important pathway for cholesterol metabolism. The gut microbiota may influence circulating cholesterol levels through its metabolites, such as bile acids, short-chain fatty acids (SCFAs), and trimethylamine-N-oxide (TMAO) (Vourakis et al., 2021; Cai et al., 2022; Xu et al., 2023).

The combination of red yeast rice, phytosterol esters and lycopene (RPL) consists entirely of food-derived ingredients. We hypothesized that there may be a close interaction between RPL and the gut microbiota, which modulates bile acid metabolism and, in turn, affects host bile acid signaling and cholesterol metabolism. High-fat, high-cholesterol (HFHC)-fed C57BL/6J mice are sensitive models for metabolic diseases. As previously mentioned, we fed C57BL/6J mice an HFHC diet to induce hypercholesterolemia (Guarino et al., 2020; Liu et al., 2022). Our aim was to investigate the efficacy and mechanisms of this dietary combination in alleviating hypercholesterolemia in mice, with a particular focus on its effects on gut microbiota and bile acid metabolism. Simvastatin (SMV), a widely prescribed statin, was used as a comparator to evaluate the relative effectiveness of the RPL combination.

## Materials and methods

### Animal model and experimental design

Male C57BL/6J specific-pathogen-free (SPF) mice, aged 6 weeks and weighing 20-21 g, were fed a HFHC diet for 12 weeks to induce obesity and hypercholesterolemia, as previously mentioned (Guarino et al., 2020; Liu et al., 2022). A total of 30 mice were randomly and equally divided into five groups: (i) normal diet only (control group), mice were fed a normal diet (TP23402, 10% of calories from fat, Trophic Animal Feed High-tech Co. Ltd., China) and received a daily gavage of the vehicle [0.5% carboxymethyl cellulose (CMC) in ddH_2_O]; (ii) HFHC diet only (HFHC group), mice were fed an HFHC diet (TP23400 mixed with 2% cholesterol, with 60% of calories from fat, Trophic Animal Feed High-tech Co. Ltd., China) and received a daily gavage of the vehicle; (iii) HFHC diet supplemented with simvastatin (SMV group): mice were fed an HFHC diet and administered 5 mg/kg/day of simvastatin (Kim et al., 2022). This dose is equivalent to 24.4 mg/day for a 60-kg adult, based on the dose conversion factor (12.3) between mice and humans (Reagan-Shaw et al., 2008) and is recognized as a recommended clinical dose (Hamoud et al., 2014); (iv) HFHC diet supplemented with a low dose of the combination (RPL1 group), mice were fed an HFHC diet and received a daily gavage of a low-dose combination containing 86.6 mg/kg RYR (Hangzhou Twin-Horse Biotechnology Co., Ltd., China), 210.8 mg/kg phytosterol ester (BASF Personal Care and Nutrition GmbH, Illertissen, Germany) and 3.1 mg/kg lycopene (Chenguang Biotech Group Co., Ltd., China); (v) HFHC diet supplemented with a high dose of the combination (RPL2 group), mice were fed an HFHC diet and received a daily gavage of a high-dose combination, which was double the dose of the RPL1 combination. The ingredient information of the diets was provided in Supplementary Table S1.

According to the reversed-phase high-performance liquid chromatography (RP-HPLC) separation and UV detector analysis of the RYR extract, the content of the active substance monacolin K in the RYR was 1.46% (Supplementary Figure S1). The level of citrinin was within the safe limit (Supplementary Table S2). The details of the assessment are presented in the Supplementary material.

Throughout the study, body weight, food intake, and glucose tolerance were monitored. Mice were fasted for 14–16 h and then euthanized to obtain blood, feces and tissues samples. The mice were purchased from Beijing Biotechnology Co., Ltd., and all animal procedures were approved by the Peking University Third Hospital Ethics Committee of Laboratory Animal (Approval No. 01301).

### Sample collection and analysis

At the end of the 8-week and 12-week intervention periods, blood samples were collected to measure serum cholesterol levels, including total cholesterol (TC), low-density lipoprotein cholesterol (LDL-C), and high-density lipoprotein cholesterol (HDL-C) using commercial kits (Nanjing Jiancheng Bioengineering Institute) (Zhou et al., 2023). Liver and intestine tissues were harvested for molecular analysis. Fecal samples were collected for 16S rRNA sequencing and targeted metabolomic profiling.

### Histopathological and molecular detection

Pancreas and epididymal fat tissues were fixed in 10% formalin, embedded in paraffin, and then 4-μm sections were cut and stained with hematoxylin and eosin (H&E) for pathological analysis.

Liver and intestine tissues were analyzed using Western blotting, quantitative polymerase chain reaction (qPCR), and immunofluorescence, as previously described (Xu et al., 2021), to assess the expression of key genes and proteins involved in cholesterol metabolism, including FXR, LDLR, and ABCG5/8. The details of each assessment are presented in the Supplementary material.

### Oral glucose tolerance test

At the end of 11th week, mice were gavaged with glucose (2.5 g/kg, dissolved in water) after a 14–16-h fast. Blood glucose levels were then measured in the tail vein using a glucose meter at 0, 15, 30, 60, 90, and 120 min (Ambati et al., 2020). The results were expressed as the area under the curve (AUC).

### Gut microbiota analysis

Fecal DNA was extracted using the DNA Extraction Kit (Qiagen). The V3–V4 region of the 16S rRNA gene was amplified and sequenced using the Illumina NovaSeq 6000 platform by OE Biotech Company (Shanghai, China). Operational taxonomic units (OTUs) were clustered, and microbial diversity was analyzed using QIIME2. The differential abundance of bacterial taxa was assessed using linear discriminant analysis (LDA) effect size (LEfSe) (Chen et al., 2022). For details, please see the Supplementary material.

### Targeted metabolomics profiling

The targeted metabolomics profiling of feces was conducted by APExBIO Technology LLC (Shanghai, China), as previously described (Zhang et al., 2025). Analyses were carried out using an ultra-high-performance liquid chromatography (UHPLC) system (1290 Infinity LC, Agilent Technologies) coupled with a QTRAP MS instrument (6500+, SCIEX). A total of up to 650 metabolites across 12 biochemical classes were detected.

Metabolites in the quality control (QC) samples with a coefficient of variation (CV) of less than 30% were considered to have reproducible measurements. A total of 482 fecal metabolites were quantified in the targeted metabolomics measurements. Any missing values for metabolites included in the analysis were treated as undetected.

### Statistical analysis

The data are presented as the mean ± standard error of mean (SEM). One-way ANOVA was employed to compare differences among the five groups, while an unpaired *t*-test was used to compare differences between two groups. Additionally, the Kruskal–Wallis test was used for the comparison of microbiota abundance. Spearman correlation was applied to assess the relationships between bile acid levels, gut bacterial genera, and metabolic parameters. Statistical analyses were performed using GraphPad Prism 8.0, and differences were considered statistically significant at a *p*-value <0.05.

## Results

### The dietary combination effectively alleviates obesity, hypercholesterolemia and impaired glucose tolerance

**Figure 1:**
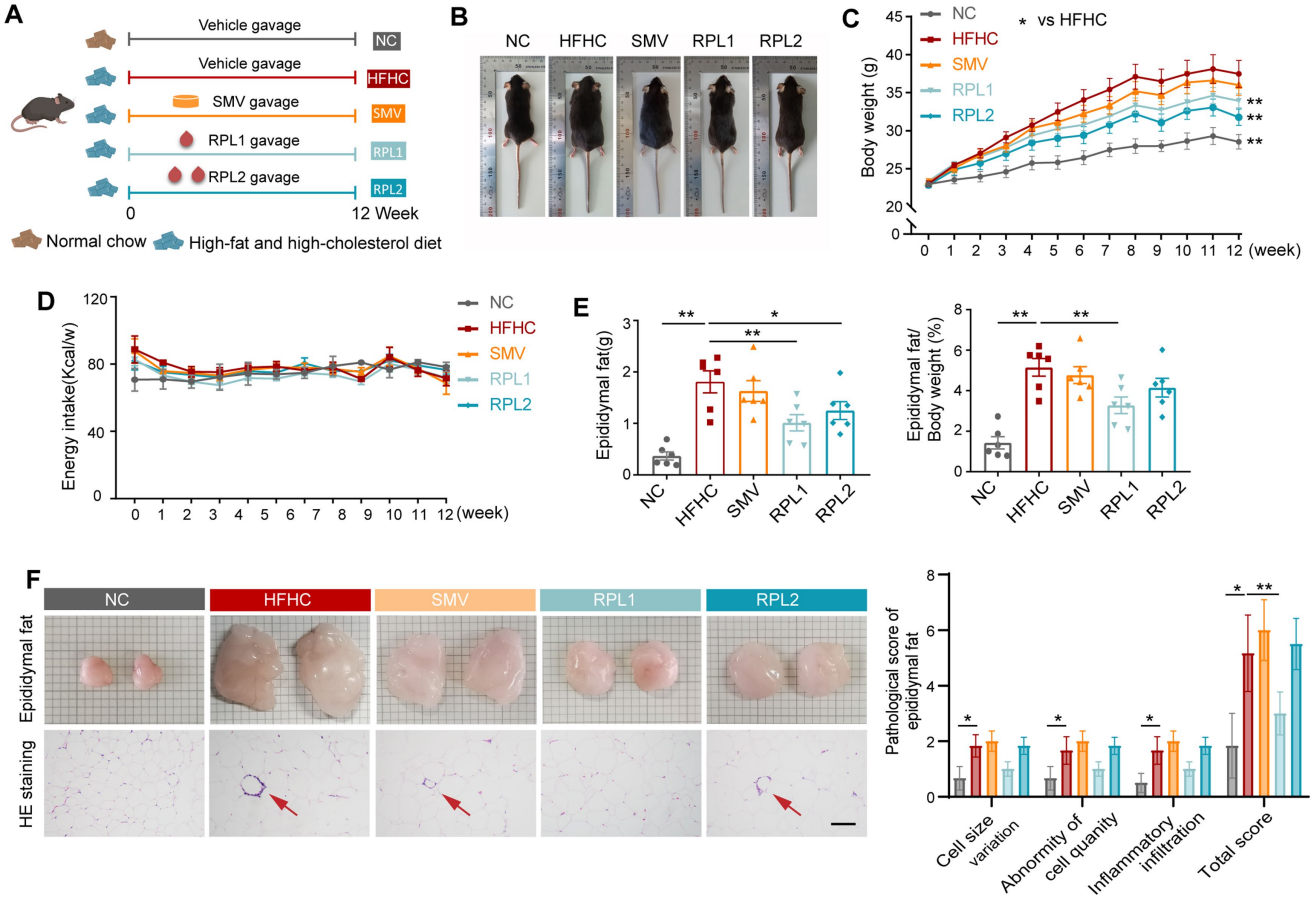
The dietary combination demonstrates superior alleviation of obesity compared to SMV. **(A)** Flowchart illustrating the mice diet and gavage intervention process. **(B)** Representative images of mice at the end of 12-week period. **(C)** Changes in body weight observed in the mice. **(D)** Energy intake recorded for the mice. **(E)** Epididymal fat weight and the percentage of epididymal fat weight relative to body weight. **(F)** Photographs, HE staining of epididymal fat, and accompanying pathological score statistics. The red arrow highlights panniculitis. For each assessment item, scores ranging from 0–4 (low to high) indicate no, slight, mild, moderate, and severe abnormalities, respectively. Scale bar: 100 μm. *N* = 6 per group. Error bars are represented by SEM, with individual data points depicted as dots. ^*^p < 0.05 and ^**^p < 0.01.

To establish a mouse model of obesity and dyslipidemia, mice were fed an HFHC diet and subjected to daily gavage with either low or high doses of RPL or SMV for a duration of 12 weeks (Figure 1A). At the conclusion of the 12-week period, mice in the HFHC group displayed significantly larger body size and epididymal fat size when compared to those in the normal chow (NC) group (Figures 1B,F). Moreover, their body weight and epididymal fat weight showed a marked increased (Figures 1C,E). HE staining revealed that the pathological scores of epididymal adipose tissue in the HFHC group were significantly higher than those in the NC group, which was characterized by abnormal cell size and increased infiltration of inflammatory cells (Figure 1F). Intervention with RPL significantly reduced body weight and epididymal fat weight in mice, without significantly affecting food or energy intake (Figures 1C–F; Supplementary Figures S2A,B). Furthermore, RPL1 intervention significantly alleviated the pathological damage to adipose tissue (Figure 1F). In contrast, SMV intervention had no significant impact on the aforementioned indices (Figures 1C–F).

**Figure 2:**
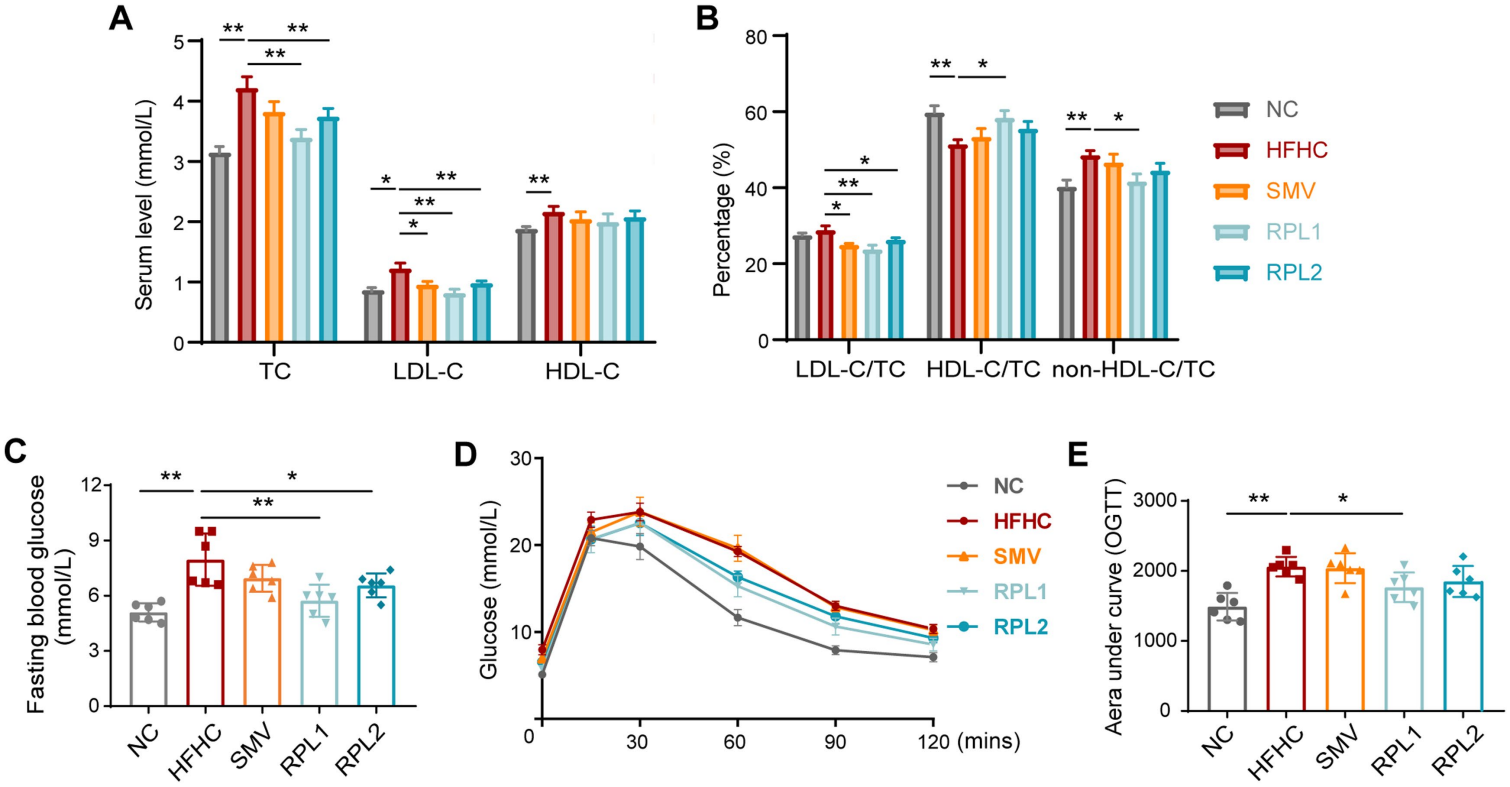
The dietary combination demonstrates superior alleviation of hypercholesterolemia and impaired glucose tolerance compared to SMV. **(A)** Serum levels of TC, LDL-C, and HDL-C at the end of the 12-week period. **(B)** The ratio of serum LDL-C, HDL-C, and non-HDL-C to TC. **(C–E)** Fasting blood glucose levels **(C)**, oral glucose tolerance test results **(D)**, and the corresponding area under the curve of (AUC) of the OGTT **(E)** for mice at the end of the 11-week period. *N* = 6 per group. Error bars are represented by SEM, with individual data points depicted as dots. ^*^p < 0.05 and ^**^p < 0.01.

As early as the 8th week, serum TC, LDL-C levels, and the ratio of LDL-C to TC were significantly elevated in the HFHC group compared to the NC group. RPL intervention partially reversed these changes (Supplementary Figure S2C). RPL1 also decreased the elevated ratio of LDL-C to TC (Supplementary Figure S2C). After 12 weeks, serum TC, LDL-C, and HDL-C levels, as well as the ratio of non-HDL-C to TC, were significantly increased in the HFHC group. Meanwhile, the ratio of HDL-C to TC was significantly decreased compared to the NC group (Figures 2A,B). RPL intervention reduced serum TC, LDL-C levels, as well as the ratio of LDL-C to TC, but had no significant effect on HDL-C levels (Figures 2A,B). Moreover, RPL1 intervention significantly reduced the ratio of LDL-C to TC and non-HDL-C to TC, and significantly increased the ratio of HDL-C to TC. SMV intervention significantly reduced the level and ratio of serum LDL-C but had no significant effect on TC levels (Figure 2A).

At 11th week, an oral glucose tolerance test (OGTT) was conducted to evaluate glucose tolerance in mice from each group. The results showed that the HFHC diet significantly increased fasting and postprandial blood glucose levels in mice compared to the NC diet (Figures 2D,E). RPL interventions significantly reduced fasting blood glucose levels in mice, and RPL1 intervention also significantly reduced postprandial blood glucose levels. In contrast, SMV intervention had no significant effect on blood glucose levels in mice (Figures 2D,E). Further HE staining of pancreatic tissue showed no significant differences in pathological damage (Supplementary Figure S2D) or the number of islets (Supplementary Figure S2E) among the five groups.

### The dietary combination intervention effectively mitigates the gut microbiota dysbiosis induced by an HFHC diet

**Figure 3:**
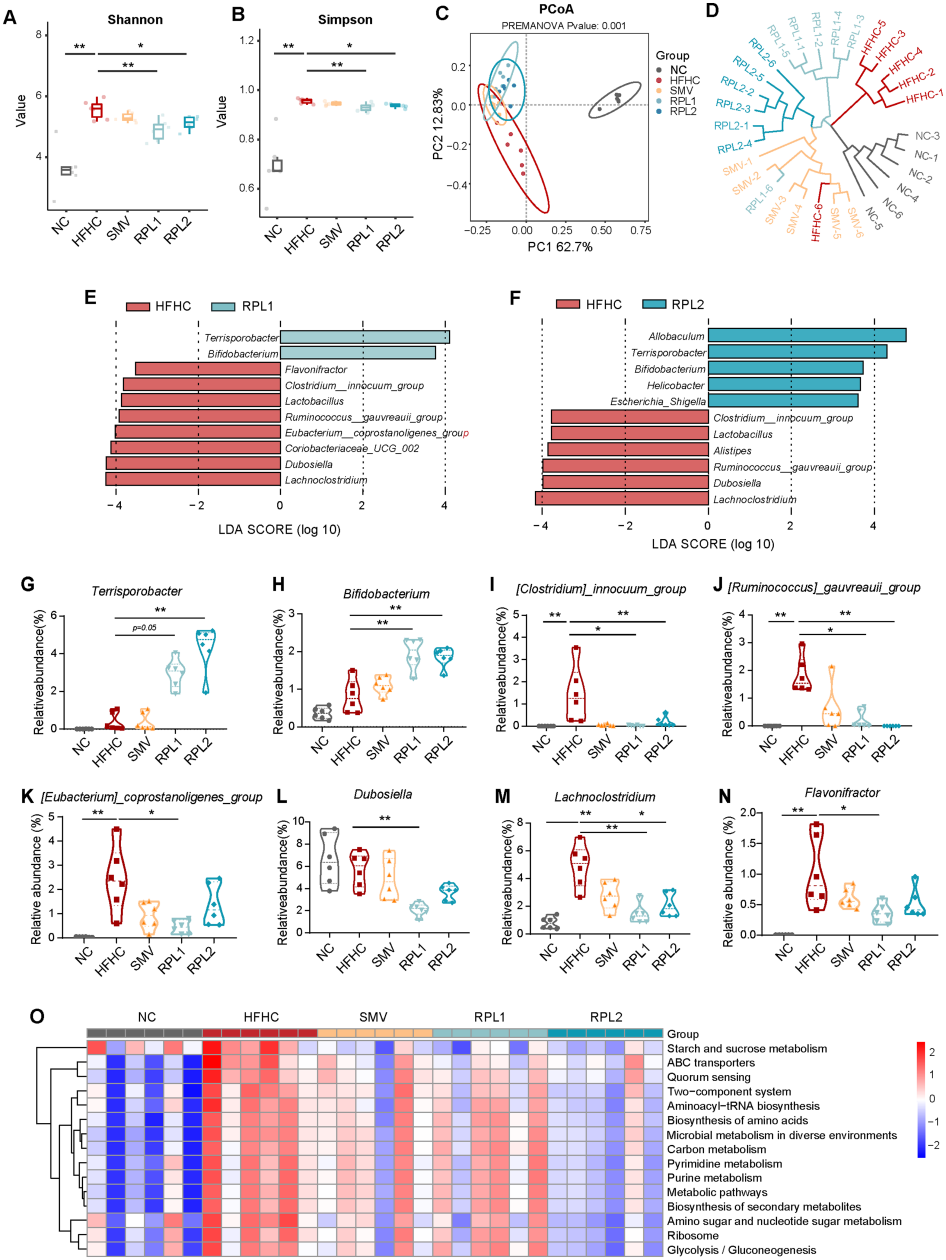
The dietary combination intervention partially mitigates the disturbance of gut microbiota dysbiosis induced by a HFHC diet. **(A,B)** The α-diversity represented by the Shannon **(A)** and Simpson **(B)** indices among the five groups. **(C,D)** The β-diversity represented by PCoA based on Bray–Curtis similarity **(C)** and UPGMA clustering based on Euclidean similarity **(D)** among the five groups. **(E,F)** Differential microbial genera identified by LEfSe analysis in the RPL1 versus HFHC groups and the RPL2 versus HFHC groups. |LDA| >3.5. **(G–N)** The relative abundance of differential gut bacterial genera (derived from **D,E**) among the five groups. **(O)** Functional enrichment analysis of differential bacteria, based on PICRUSt2 among the five groups. *N* = 6 per group. Data are expressed as mean ± SEM, ^*^p < 0.05 and ^**^p < 0.01. UPGMA, unweighted pair group method with arithmetic mean; LDA, linear discriminant analysis score.

16S rRNA gene sequencing revealed that RPL interventions significantly counteracted the increase in α-diversity, as indicated by the Shannon and Simpson indices, which were elevated in the HFHC group (Figures 3A,B). An analysis of β-diversity showed that the gut microbiota of mice fed an HFHC diet exhibited marked differences from that of mice on a control diet, and RPL interventions partially restored these microbial communities to a more normal state (Figures 3C,D). In contrast, the SMV intervention had no significant impact on the α-diversity of the gut microbiota compared to the HFHC group. Moreover, the clustering pattern of the gut microbiota in the SMV group, as shown by β-diversity analysis, closely resembled that of the HFHC group (Figure 3C).

Subsequently, LEfSe analysis was performed to pinpoint the changes in gut bacterial genera following RPL interventions (Figures 3E,F). The results showed that *Terrisporobacter* (Figures 3E–G) and *Bifidobacterium* (Figures 3E,F,H) were more abundant in the RPL groups than in the HFHC group. Additionally, several bacterial genera that were enriched in the HFHC group showed a decrease in abundance after RPL interventions. These included *Clostridium_innocuumm_group*, *Ruminococcus_gauvreauii_group*, *Eubacterium_coprostanoligens_group*, *Lachonoclostridium* and *Dubosiella* (Figures 3E,F,I–M). Furthermore, the RPL1 intervention led to a significant reduction in the abundance of *Coriobacteriaceae_UCG_002* (Figure 3E) and *Flavonifrator* (Figures 3E,N) compared to the HFHC group. Functional prediction analysis across the five groups showed that the RPL intervention reversed a number of metabolic pathways that were upregulated by the HFHC diet. These pathways included starch and sucrose metabolism, amino acid synthesis, carbon metabolism, secondary metabolite synthesis, amino sugar and nucleotide sugar metabolism, and glycogen synthesis (Figure 3O).

### The dietary combination intervention partially reverses the changes in fecal metabolites induced by an HFHC diet

**Figure 4:**
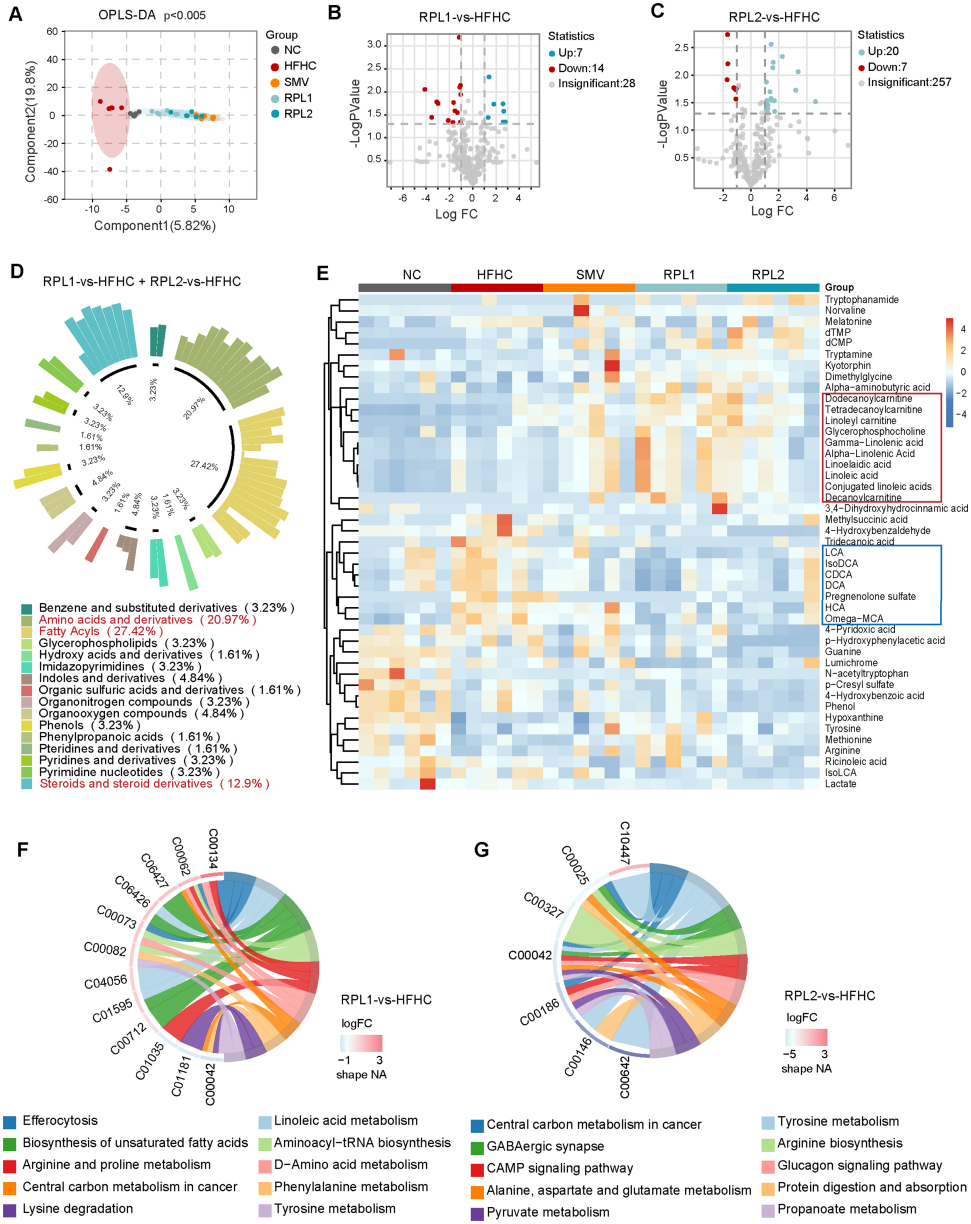
The dietary combination intervention partially restores fecal metabolite changes induced by an HFHC diet in mice. **(A)** Structural analysis of fecal metabolites using OPLS-DA among the five groups. **(B,C)** Volcano plots showing differential metabolites after low **(B)** or high **(C)** doses of RPL intervention, compared with the HFHC group. **(D,E)** Classification **(D)** and abundance **(E)** of differential metabolites following low and high doses of RPL intervention, compared with the HFHC group. The red and blue boxes marked metabolites that were upregulated and downregulated, respectively, after RPL intervention. **(F,G)** Significantly altered pathways after low **(F)** or high **(G)** doses of RPL intervention, compared with the HFHC group. *N* = 6 per group.

To identify the metabolites affected by RPL intervention in mouse metabolism, we carried out a targeted metabolomics analysis on fecal samples. The OPLS-DA analysis comparing the five groups revealed a distinct clustering pattern in the HFHC group as opposed to the control group. Moreover, both RPL and SMV interventions brought about alterations in the clustering of fecal metabolites when compared with the HFHC group (Figure 4A).

Subsequently, we investigated the changes in fecal metabolites following RPL interventions (fold change >2, *p* < 0.05). The volcano plots showed that the RPL1 intervention led to the upregulation of seven metabolites and the downregulation of 14 metabolites in feces (Figure 4B). In contrast, the RPL2 intervention resulted in the upregulation of 20 metabolites and the downregulation of seven metabolites (Figure 4C). The classification circle plot demonstrated a total of 45 differential metabolites after low- and high-doses RPL interventions. These metabolites primarily belonged to the categories of fatty acyls, amino acids and their derivatives, as well as sterols and their derivatives (Figure 4D). The heatmap displayed the relative abundance of these 45 differential metabolites across the five groups (Figure 4E). Additionally, functional enrichment analysis revealed that the pathways significantly altered after the RPL1 intervention included unsaturated fatty acid synthesis and oleic acid metabolism (Figure 4F). On the other hand, the pathways significantly affected after the RPL2 intervention encompassed pyruvate metabolism, the glycogen signaling pathway, and protein digestion and absorption (Figure 4G).

**Figure 5:**
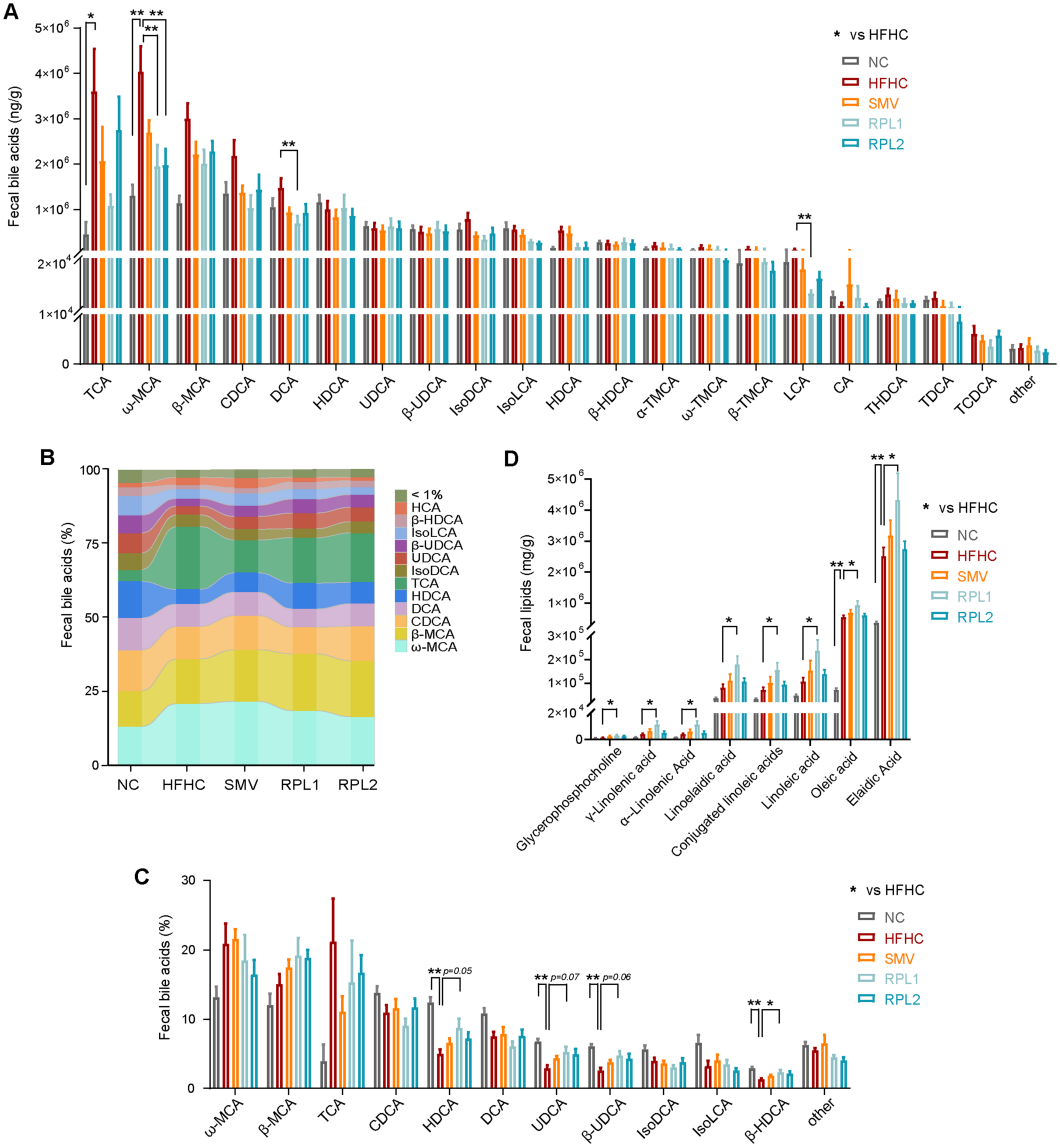
The dietary combination intervention alters bile acid and lipid components in feces. **(A)** Levels of the top 20 fecal bile acids (BA). **(B,C)** Proportion of different bile acid components in feces. **(D)** Levels of lipid components in feces. *N* = 6 per group. Data are expressed as mean ± SEM, ^*^p < 0.05 and ^**^p < 0.01.

To evaluate the levels of specific bile acids after the intervention, we compared the fecal bile acid components among the five groups. A total of 27 fecal bile acids were measured, and the data for the top 20 bile acids are presented below. The RPL1 intervention notably decreased the elevated levels of ω-MCA, DCA and LCA in the HFHC group. Meanwhile, the RPL2 intervention also significantly reversed the increase in ω-MCA levels (Figure 5A).

The stacked bar chart (Figure 5B) and histogram (Figure 5C) demonstrated that RPL1 intervention increased the proportions of HDCA (*p* = 0.05), UDCA (*p* = 0.07), β-HDCA (*p* = 0.06), and β-UDCA (*p* < 0.05) in the feces compared to the HFHC group. Furthermore, we compared fecal lipid excretion after RPL or SMV intervention. The results showed that the RPL1 intervention significantly increased the fecal excretion of long-chain fatty acids compared to the HFHC group, while the RPL2 and SMV interventions had no such effect (Figure 5D).

### The dietary combination intervention effectively activates the FXR signaling pathway in the liver

**Figure 6:**
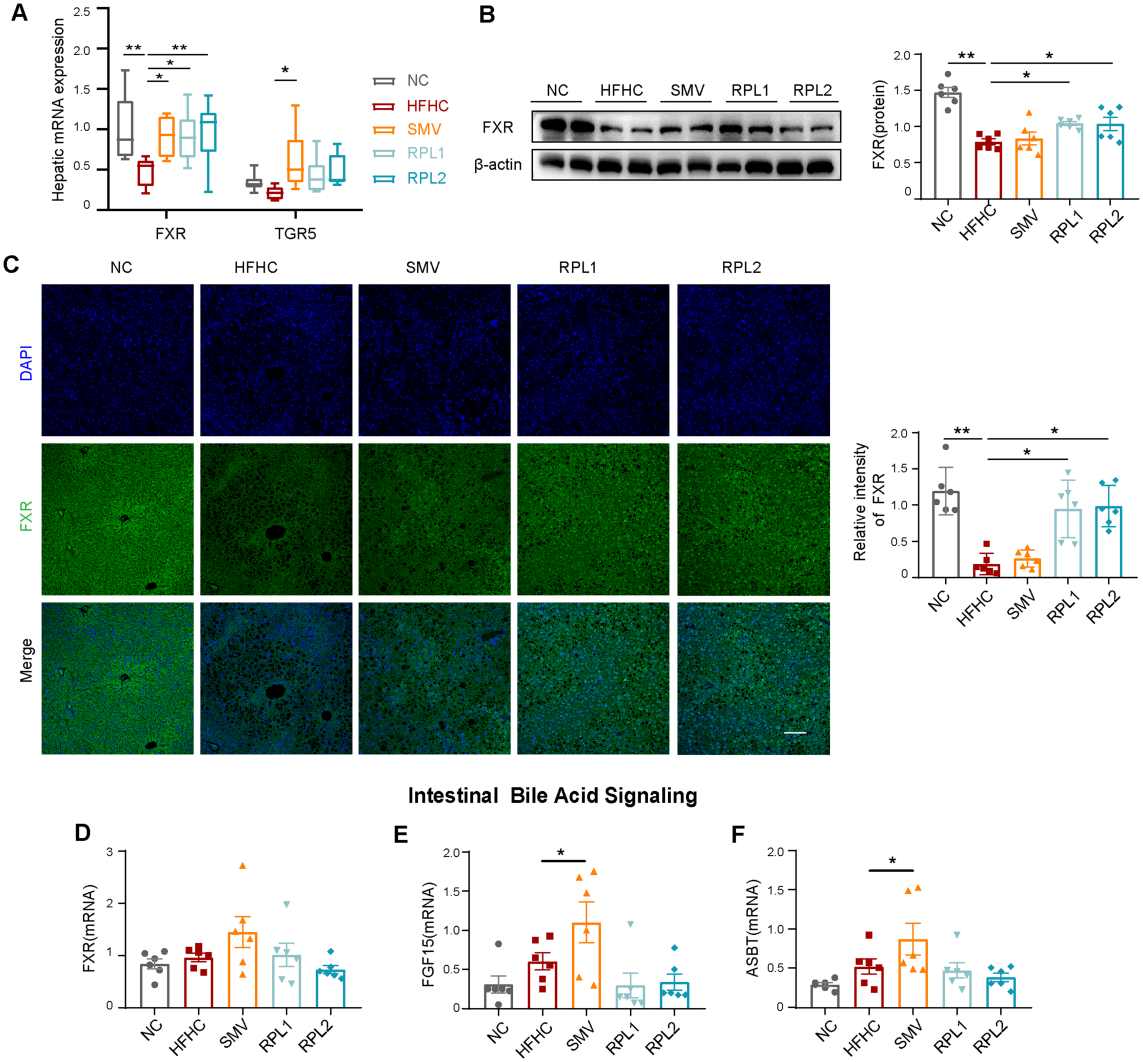
Intervention with the combination activates the FXR signal in the liver. **(A)** mRNA expression of canonical bile acid receptors in the liver. **(B)** Western blot images of hepatic FXR and the corresponding statistical analysis. **(C)** Immunostaining of hepatic FXR and the corresponding statistical analysis. Scale bar, 100 μm. **(D–F)** mRNA expression levels of intestinal FXR **(D)**, FGF15 **(E)**, and ASBT **(F)**. *N* = 6 per group. Error bars are represented by SEM, with individual data points depicted as dots. ^*^p < 0.05 and ^**^p < 0.01.

FXR and Takeda G protein-coupled receptor 5 (TGR5) are well-established as canonical bile acid receptors (Fleishman and Kumar, 2024). Our results showed that the HFHC diet significantly decreased both the mRNA and protein levels of hepatic FXR. Notably, these reductions were substantially reversed by the intervention with the RPL (Figures 6A,B). Immunostaining further corroborated the upregulation of the hepatic FXR protein level following RPL intervention compared to the HFHC group (Figure 6C).

The genes involved in bile acid synthesis are under dual regulation by the hepatic FXR signaling pathway and the intestinal FXR-fibroblast growth factor 15 (FGF15) axis. The apical sodium-dependent bile acid transporter (ASBT) plays a crucial role in the reabsorption of approximately 95% of bile acids in the intestine. Our findings demonstrated that RPL intervention did not exert a significant influence on the expression of components within the FXR-FGF15 signaling pathway or on the expression of the bile acid transporter ASBT in the intestine (Figures 6D–F). Likewise, when compared to the HFHC group, RPL intervention had no remarkable effect on the expression of downstream bile acid synthesis genes in the liver (Supplementary Figures S3A–D). Moreover, there were no significant differences in the levels of total bile acid (TBA) in either the serum or the liver among the five experimental groups (Supplementary Figures S3E,F).

### The dietary combination intervention promotes the expression of genes involved in cholesterol uptake and excretion within the liver

**Figure 7:**
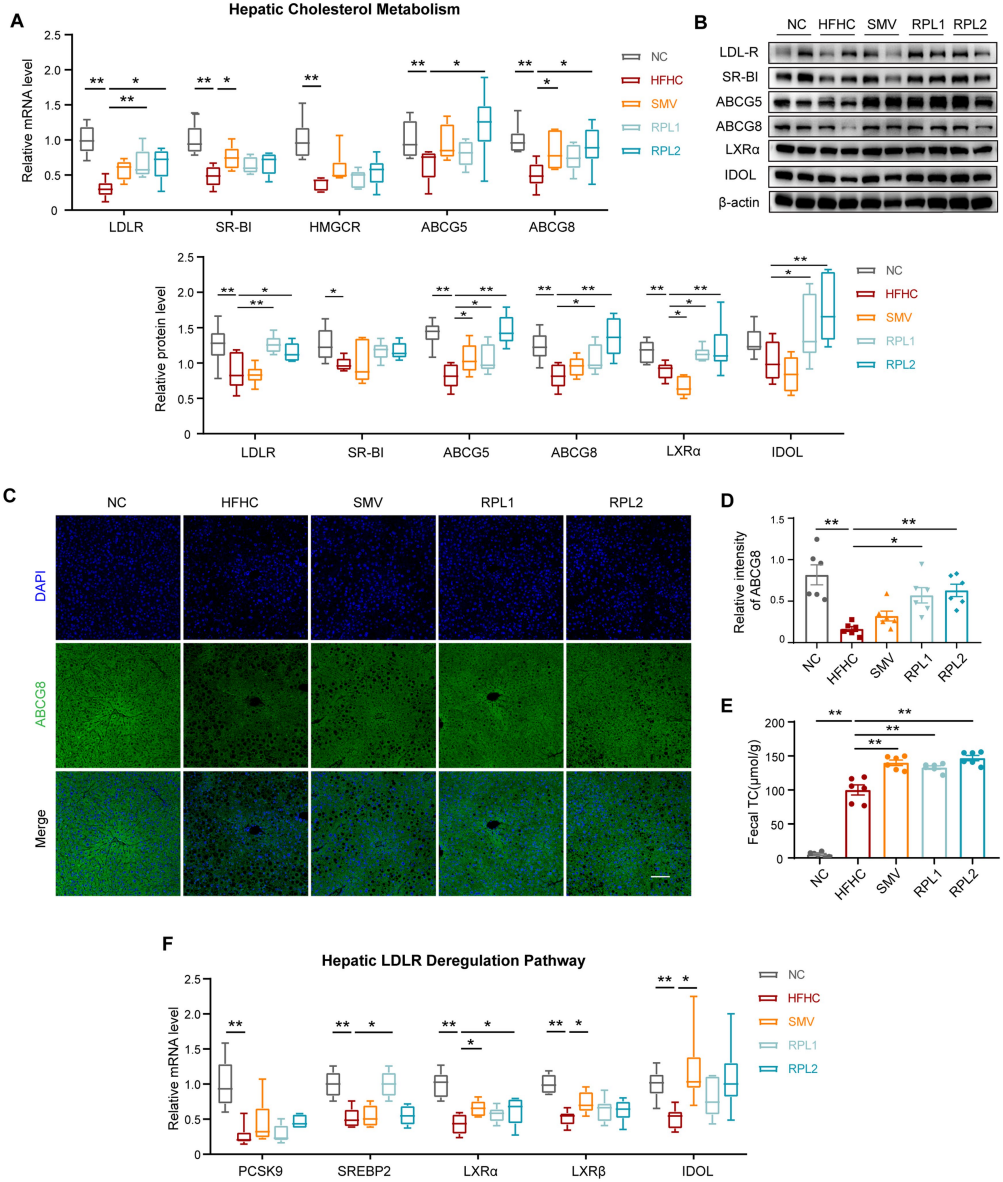
The dietary combination intervention promotes the expression of cholesterol uptake and excretion genes in the liver. **(A)** mRNA expression levels of hepatic cholesterol metabolism-related genes, including LDLR, SR-BI, HMGCR and ABCG5/8. **(B)** Chemiluminescence images of hepatic LDLR, SR-BI, ABCG5/8, LXRα and IDOL, along with the corresponding statistical analysis. **(C)** Immunostaining of hepatic ABCG8, with the corresponding statistical analysis shown. **(D)** mRNA expression levels of genes related to LDLR degradation, including SREBP2, PCSK9, LXRα, LXRβ, and IDOL. **(E)** Levels of fecal TC. **(F)** mRNA expression levels of intestinal cholesterol transport-related genes, including NPC1L1, ABCA1, ABCG1, and ABCG5/8. *N* = 6 per group. Error bars are represented by SEM, with individual data points depicted as dots. ^*^p < 0.05 and ^**^p < 0.01.

We employed a combination of immunofluorescence, RT-qPCR, and Western blot techniques to evaluate the expression of cholesterol metabolism genes. In the liver, the LDLR and scavenger receptor class B type I (SR-BI) are crucial for the uptake of LDL-C and HDL-C from the bloodstream by hepatocytes, respectively. ABCG5 and ABCG8 form a heterodimer that plays a pivotal role in facilitating the excretion of excess cholesterol from hepatocytes into bile. HMGCR functions as a key enzyme in the endogenous synthesis of cholesterol. Our results clearly demonstrated that the RPL interventions significantly counteracted the reductions in the hepatic levels of LDLR, ABCG5, and ABCG8. However, when compared to the HFHC group, these interventions had no significant impact on the decreased levels of hepatic SR-BI and HMGCR (Figures 7A–C).

To determine whether the increase in LDLR expression was due to a reduction in degradation signals, we detected these two degradation pathways using RT-qPCR and Western blot. The results showed that, in comparison to the HFHC group, neither of the degradation signals showed a decrease following RPL interventions (Figure 7D).

In the intestine, Niemann-Pick C1-like 1 (NPC1L1), ATP-binding cassette sub-family A member 1 (ABCA1), and ATP-binding cassette sub-family G member 1 (ABCG1) act as transporters for cholesterol absorption, while ABCG5/8 form a transporter complex responsible for cholesterol excretion into the intestinal lumen. Our findings revealed that RPL interventions had no significant effect on the expression of these cholesterol transporters in the intestine (Figure 7F).

Furthermore, an analysis of the TC level in mouse feces confirmed that RPL intervention further augmented the increased TC levels observed in the HFHC group (Figure 7E). Taken together, these results strongly suggest that RPL intervention upregulates the expression of cholesterol transporters and promotes cholesterol excretion in the liver.

### The alterations in bile acids profiles following the intervention with the dietary combination exhibit a significant correlation with changes in the gut microbiota

**Figure 8:**
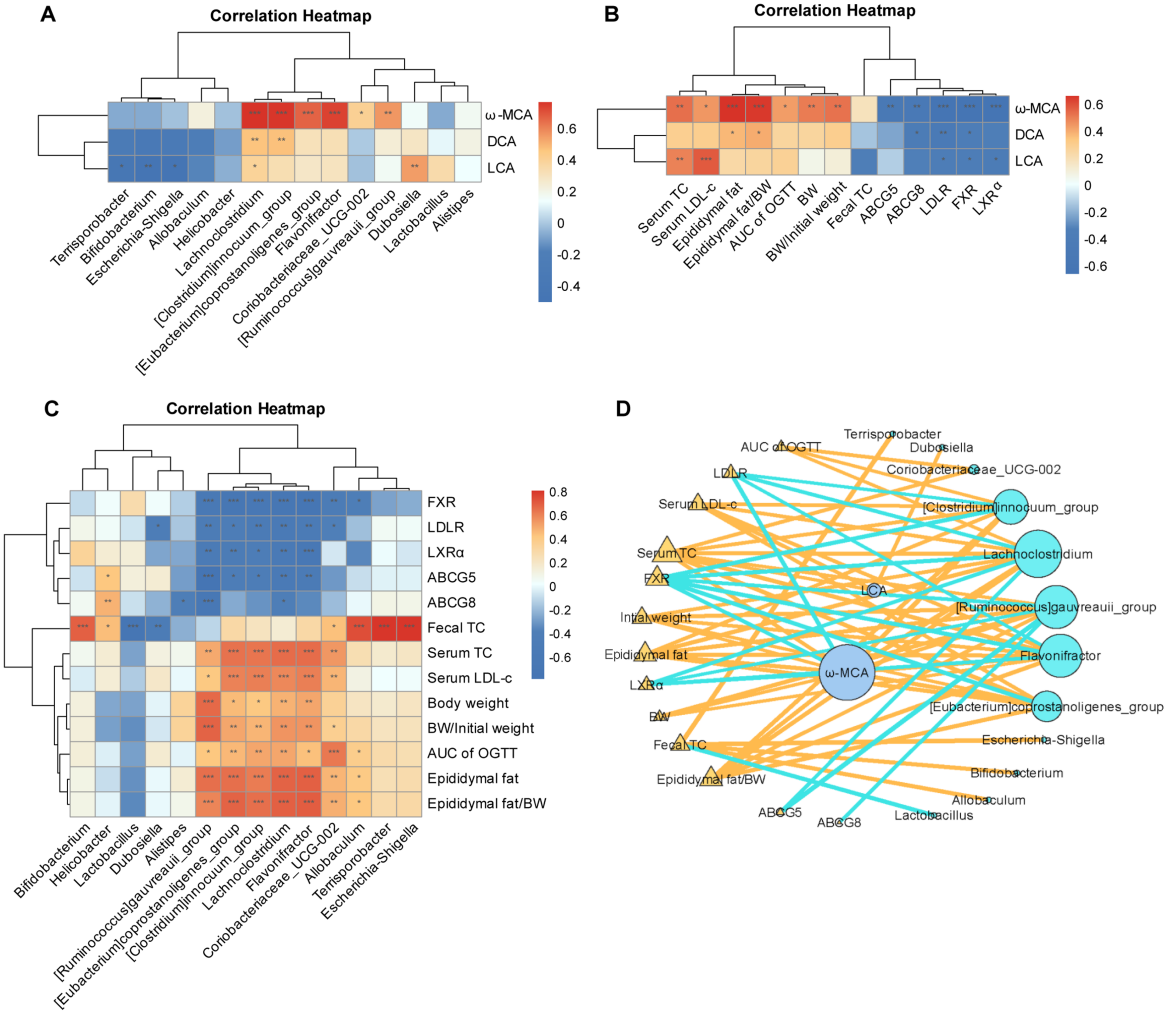
The changes in bile acids following the intervention with the combination exhibit a significant correlation with gut microbiota. **(A–C)** Spearman’s correlation analysis between the concentrations of differential bile acids and differential bacterial genera induced by the RPL intervention **(A)**, between the differential bile acid levels induced by the RPL intervention and metabolic parameters **(B)**, between the differential bacterial genera induced by the RPL intervention and metabolic parameters. **(D)** Network interaction analysis among bile acid levels, gut bacterial genera, and metabolic parameters. *N* = 6 per group. Individuals from the five groups were all included.

We employed Spearman’s correlation analysis to thoroughly investigate the potential associations among fecal bile acid alterations, gut microbiota changes, and various metabolic parameters. The analysis yielded several noteworthy findings. Firstly, it was revealed that *Bifidobacterium* and *Terisporobacter*, both of which were enriched by the RPL intervention, exhibited a significant negative correlated with the levels of all three differential bile acids (Figure 8A). In addition, the *Clostridium_innocuumm_group*, *Eubacterium_coprostanoligens_group*, *Lachnoclostridium*, *Flavonifrator*, *Coriobacteriaceae_UCG*-*002*, and *Ruminococcus_gauvreauii_group* all exhibited a significant positive correlation with the level of ω-MCA (Figure 8B). Moreover, these bacterial groups also showed a significant positive correlation with obesity and glycolipid-related parameters (Figure 8C).

Furthermore, ω-MCA was found to have a significant positive correlation with mouse obesity and glycolipid parameters. Conversely, ω-MCA exhibited a significant negative correlation with bile acid signaling and cholesterol transporter levels (Figure 8D). The network interaction map further elucidated the complex interplay among the differential bacterial genera, the three bile acids under investigation, and the metabolic parameters. Notably, ω-MCA occupied a central position in this network, highlighting its potential importance as a key mediator in the interactions between the gut microbiota, bile acid metabolism, and metabolic health.

## Discussion

Our study is the first to demonstrate that the dietary combination of RYR, phytosterol ester, and lycopene exerts significant effects in reducing obesity and hypercholesterolemia in mice fed a high-fat, high-cholesterol (HFHC) diet. This combination was found to alter cholesterol metabolism, gut microbiota composition, and bile acid signaling. The altered bile acids profile further activated the hepatic FXR-LDLR/ABCG5/8 signaling pathway, which promoted hepatic cholesterol excretion and reduced serum cholesterol levels.

It is noteworthy that the RPL combination proved to be more effective in alleviating metabolic disorders compared to simvastatin. The RPL combination reduced LDL-C levels by 33% at the low dose and 20% at the high dose, whereas simvastatin achieved a 22% reduction. Moreover, both doses of RPL significantly reduced TC levels, alleviated obesity, and decreased fasting blood glucose levels in mice, effects that were not observed with simvastatin. Interestingly, when comparing the two-dose RPL groups, the low-dose group was more effective in reducing serum cholesterol and blood glucose levels than the high-dose group.

16S rRNA gene sequencing revealed a close interaction between the RPL combination and the gut microbiota. In our study, the HFHC diet increased the α-diversity of the gut microbiota, a finding consistent with some previous studies (Wang et al., 2020; Duan et al., 2021; Guo et al., 2021) but in contrast to others that reported decreased α-diversity (Wang et al., 2023; Zhou et al., 2023). The RPL intervention significantly reversed the increased diversity induced by the HFHC diet, while simvastatin had no such effect. Additionally, β-diversity analysis showed that the microbial structure of the RPL groups was distinct from that of the HFHC group, whereas the structure of the simvastatin group almost overlapped with that of the HFHC group. These findings confirm the unique ability of the RPL combination to partially reverse HFHC-induced dysbiosis, a benefit not provided by simvastatin.

Interestingly, we found no significant differences between the low-dose (RPL1) and high-dose (RPL2) groups for most results. To explore the dose-effect relationship of the dietary combination, we conducted experiments with gradient doses. In addition to RPL1 and RPL2, we also included RPL3, which had a dose double that of RPL2. Surprisingly, while RPL3 alleviated obesity (Supplementary Figures S4A–D), it failed to improve serum cholesterol parameters (Supplementary Figures S4E–G). Gut microbiota analysis revealed divergent dose effects. Both RPL1 and RPL2 significantly reversed the increased α-diversity induced by the HFHC diet, while RPL3 did not (Supplementary Figures S4H,I). Moreover, β-diversity analysis demonstrated distinct clustering for RPL1 and RPL2 but a near-complete overlap between RPL3 and HFHC groups (Supplementary Figure S4J). These results demonstrate that different doses of the RPL combination have varying impacts on the gut microbiota. Increasing the administered dose did not improve cholesterol-lowering effectiveness and may potentially compromise its efficacy through gut microbiota modulation. Taken together, these results provided critical guidance for optimal dosage selection.

The RPL intervention increased the abundance of several probiotics, including *Bifidobacterium*. *Bifidobacterium* belongs to the Actinobacteria phylum and is a beneficial bacterium in the gut. In patients and mouse models with hypercholesterolemia, *Bifidobacterium* is negatively correlated with serum total cholesterol, and it may reduce cholesterol levels by regulating the gut microbiota (Zhang et al., 2021; Kou et al., 2023). Additionally, it has been reported that *Bifidobacterium* can metabolize bile acids to produce HDCA (Zhang et al., 2024), and an increased proportion of HDCA has been reported to reduce intestinal cholesterol absorption by weakening the emulsifying ability of lipids (van de Heijning et al., 1994; Wang et al., 2003). Consistent with these studies, our fecal metabolome data showed increases in the proportion of HDCA and fecal lipid excretion after RPL1 intervention.

Studies have reported increased abundances of *Clostridium* (Iqbal et al., 2025), *Ruminococcus* (Hefni et al., 2025; Nie et al., 2025) and *Lachnoclostridium* (Cai et al., 2022; Zhang et al., 2023; Yang et al., 2025) in patients and mouse models with hypercholesteremia, and obesity, and the same results were observed in our experiments (Figures 3I,M). Importantly, the RPL intervention markedly decreased the elevated levels of *Clostridium*, *Ruminococcus* and *Lachnoclostridium* (Figures 3I,M). Correlation analysis also indicated that these bacterial genera were positively associated with hyperglycemia, hypercholesteremia, and obesity (Figure 8A). These results suggest that the RPL combination can suppress the growth of potentially harmful bacteria, thereby improving obesity and hypercholesteremia.

The deconjugation and transformation of bile acids in the intestinal lumen are entirely dependent on the gut microbiota. In mice, *Clostridium*, *Ruminococcus*, and *Eubacterium* participate in the C-7 epimerization and 7α-dehydroxylation of bile acids, metabolizing β-MCA to generate ω-MCA (Jia et al., 2018). *Clostridium* has also been reported to promote the conversion of CA and CDCA to DCA and LCA in the intestinal lumen, respectively (Jia et al., 2018). In our study, the RPL intervention caused a reduction in *Clostridium*, *Ruminococcus*, and *Eubacterium*. Consistently, our fecal metabolomics data demonstrated a significant reduction in ω-MCA, DCA and LCA after RPL intervention. These results suggested that the RPL intervention may affect bile acid levels by regulating the gut microbiota.

Bile acid components exhibit different affinities for bile acid receptors. It has been reported that MCAs have an antagonistic effect on FXR signals (Sayin et al., 2013; Gonzalez et al., 2016; Fu et al., 2019). We then detected FXR in mice, and the results showed that the RPL intervention reversed the decreased hepatic FXR signal but had no significant effect on intestinal FXR compared to the HFHC group. A previous study has shown that HDCA administration can inhibit intestinal FXR while activating hepatic FXR signal, thereby improving lipid metabolism (Kuang et al., 2023). In our model, although the content of the FXR-antagonistic bile acid MCA decreased in feces, the proportion of HDCA increased, which may locally inhibit FXR in the intestine. We postulate that as a result, the intestinal FXR signaling was not significantly changed.

Activation of FXR promotes the stability of LDLR mRNA and upregulates its expression level (Singh et al., 2018). In rats and humans, FXR upregulation induces the expression of proteins ABCG5 and ABCG8, which are responsible for cholesterol excretion (Yu et al., 2005; El Kasmi et al., 2021). Therefore, we next detected the expression of cholesterol metabolism genes in the liver. Surprisingly, the RPL intervention significantly reversed the reduction in hepatic LDLR, ABCG5, and ABCG8 transporters caused by the HFHC diet but had no significant effect on cholesterol synthesis genes and HDL-C transporters.

Although the LDLR level increased after the RPL intervention, its main ubiquitination and lysosomal degradation pathways did not decrease. This indicates that the increase in LDLR expression level was not due to reduced degradation and that LDLR may be directly induced by FXR activation. Consistent with these results, the total cholesterol level in feces was found to increase significantly after the RPL intervention compared to the HFHC group. Taken together, our results indicate that the RPL intervention significantly upregulated hepatic uptake of LDL-C from the bloodstream and secretion of cholesterol into the bile by activating the hepatic FXR-LDLR/ABCG5/8 pathway.

We observed that the regulation of the gut microbiota on bile acid metabolism is a key mechanism for the RPL intervention. However, the specific role of altered gut microbiota and metabolites in ameliorating hypercholesterolemia requires further validation. Moreover, clinical trials are warranted to verify the effects of the RPL intervention in patients with hypercholesterolemia.

In summary, this study revealed that the RPL combination remodeled the gut microbiota and bile acid metabolism to activate the hepatic FXR-LDLR/ABCG5/8 pathway, thereby increasing hepatic cholesterol excretion and reducing serum cholesterol levels. Among the groups, RPL1 was found to be more effective in reducing serum cholesterol and serum glucose levels than RPL2. The RPL combination may serve as a more effective strategy with fewer adverse effects for the clinical management of hypercholesterolemia caused by a HFHC diet.

## Data Availability

The original contributions presented in the study are publicly available. This data can be found here: https://www.ncbi.nlm.nih.gov/bioproject/PRJNA1295338/ accession number: PRJNA1295338.
