# Supplementary material for: A dietary combination of red yeast rice, phytosterol ester and lycopene ameliorates hypercholesterolemia by regulating gut microbiota and activating hepatic FXR-LDLR/ABCG pathway in mice
Source: Front Microbiol. 2025 Aug 7;16:1622818. doi: 10.3389/fmicb.2025.1622818 (PMC12369415; doi:10.3389/fmicb.2025.1622818)
Supplement: Supplementary file 1 [file Data_Sheet_1.docx]

**A Dietary Combination of Red Yeast Rice, Phytosterol Ester and Lycopene Ameliorates Hypercholesterolemia by Regulating Gut Microbiota and Activating Hepatic FXR-LDLR/ABCG Pathway in Mice**

Jingxian Xu^1 2^, Xin Huang^1 2^, Fei Pei^3^, Yuzhu Chen^1 2^, Cunzheng Zhang^1 2^, Lingling Zhao^4^, Hua Zhang^4^, Jindong Zhang*^1 2^, Liping Duan* ^1 2^

^1^ Department of Gastroenterology, Peking University Third Hospital, Beijing, China

^2^ Beijing Key Laboratory for *Helicobacter pylori* Infection and Upper Gastrointestinal Diseases, Beijing, China

^3^ Department of Pathology, Peking University School of Basic Medical Sciences, Peking University Third Hospital, Peking University Health Science Center, Beijing, China

^4^ H&H Group, H&H Research, China Research and Innovation Center, Guangzhou, China

^*^ Corresponding author:

Liping Duan, MD; E-mail: [duanlp@bjmu.edu.cn](mailto:duanlp@bjmu.edu.cn)

Jindong Zhang, MD; E-mail: zhangjd@bjmu.edu.cn

**This file includes:**

Supplementary Figure S1 to S4

Supplementary Table S1 to Table S3

Supplementary Information for Materials and methods

**Table S1.** The ingredient information of the diets.

| **Ingredients** | **TP 23400(g/kg)** | **TP 23402(g/kg)** |
| --- | --- | --- |
| Protein (casein, L - cystine) | 201 | 141 |
| Carbohydrate (dextrin, sucrose, corn starch) | 320 | 722 |
| Fat (soybean oil, lard) | 341 | 40 |
| Fiber (Cellulose) | 71 | 50 |
| Mineral and vitamin mixture | 67 | 47 |
| Antioxidant (TBHQ) | 0.066 | 0.008 |
| **Total** | **1000** | **1000** |
| Energy, Kcal/g | 5.1 | 3.6 |
| % Kcal from - Protein | 14% | 14% |
| % Kcal from - Carbohydrate | 26% | 76% |
| % Kcal from - Fat | 60% | 10% |
| **% Kcal from - Total** | **100%** | **100%** |


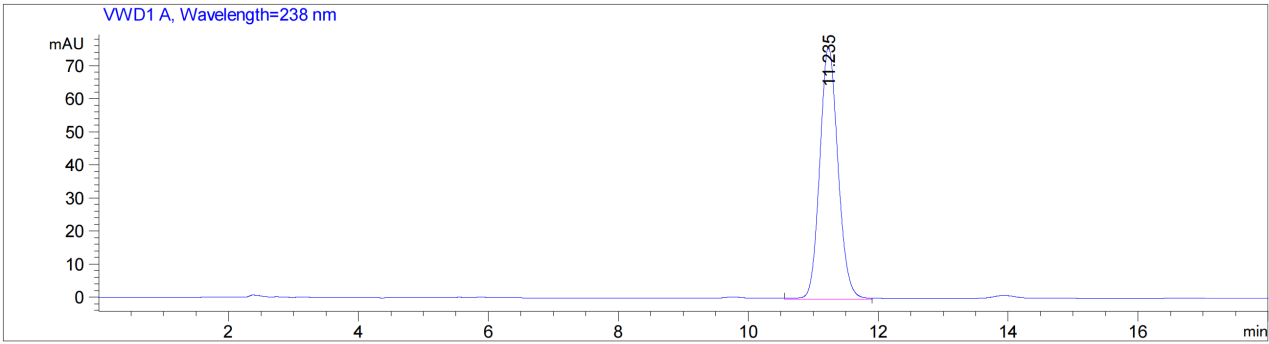


**A**


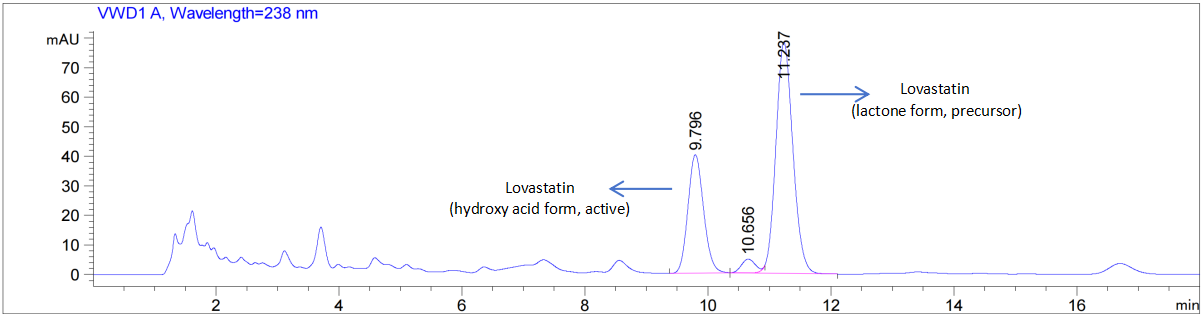


**B**

**Fig. S1.** Quantification of Monacolin K (Lovastatin) in Red Yeast Rice.

(A) Spectrum peak plot of Lovastatin standard.

(B) Spectrum peak plot of of red yeast rice.

**Table S2.** The active substance monacolin K and other quality control information in red yeast rice.

| ITEM | STANDARD | SPECIFICATION | RESULT |
| --- | --- | --- | --- |
| Monacolin K (%) | QB/T 2847 | 1.3 - 1.6 | 1.46 |
| Appearance | QB/T 2847 | up to the standard | up to the standard |
| Particle Size (%) 120 Mesh | ChP(2020) | ≥95 | 98 |
| Moisture (%) | GB 5009.3 | ≤10 | 3.69 |
| Citrinin (μg/kg) | GB 5009.222 | ≤50 | ＜50 |
| Pb (mg/kg) | GB 5009.12 | ≤1.0 | ＜1.0 |
| As (mg/kg) | GB 5009.11 | ≤1.0 | ＜1.0 |
| Hg (mg/kg) | GB 5009.17 | ≤0.3 | ＜0.1 |
| Aflatoxin B1 (μg/kg) | GB 5009.22 | ≤5 | ＜5 |
| Total Bacterial Count (CFU/g) | GB 4789.2 | ≤5000 | 15 |
| Coliform /(MPN/g) | GB 4789.3 | ≤0.3 | ＜0.3 |
| Mold (CFU/g) | GB 4789.15 | ≤25 | 5 |
| Yeast (CFU/g) | GB 4789.15 | ≤25 | ＜10 |
| Salmonella | GB 4789.4 | Negative | Negative |
| Staphylococcus aureus | GB 4789.10 | Negative | Negative |


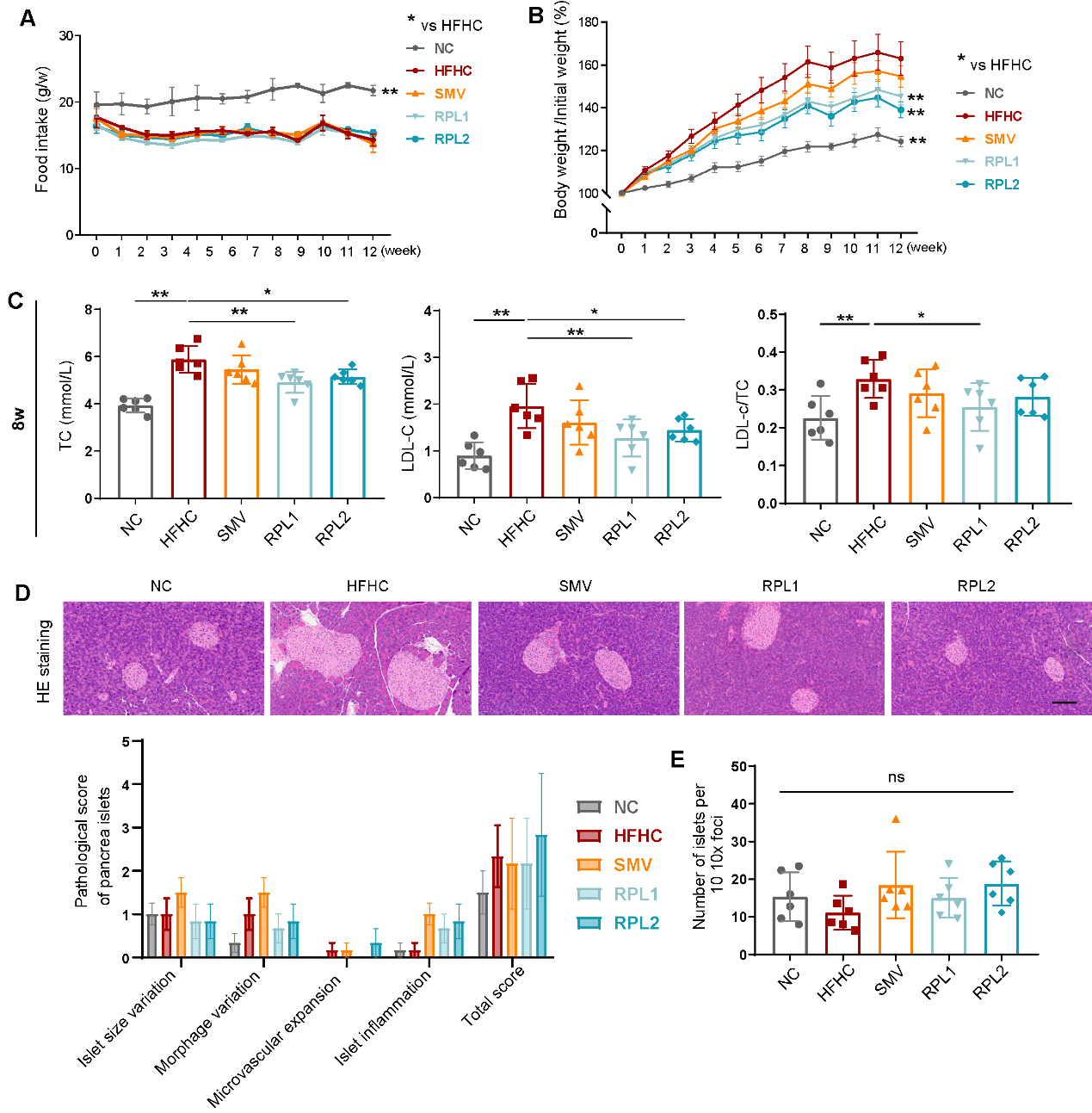


**Fig. S2.** The impact of red yeast rice formula on serum cholesterol levels and pancreas islets. (A) Food intake recorded for the mice. (B) Percentage change in body weight relative to the initial weight of the mice. (C) Serum levels of TC, LDL-C, and HDL-C at the end of the 8w period. (D) HE staining of the pancreas, along with corresponding pathological score statistics. For each assessment item, scores ranging from 0-4 (low to high) indicate no, slight, mild, moderate, and severe abnormalities, respectively. Scale bar: 100μm. (E) Count of pancreas islets. N=6 per group. Error bars are represented by SEM, with individual data points depicted as dots. *p < 0.05, **p < 0.01.


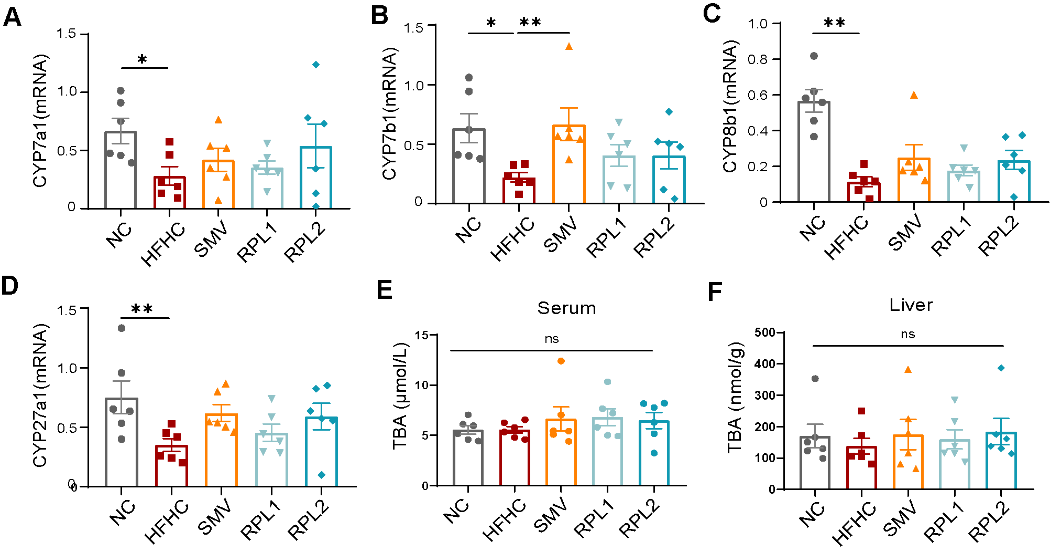


**Fig. S3.** Expression levels of bile acid synthesis gene and total bile acid concentrations. (A-D) mRNA expression levels of hepatic bile acid synthetic genes. (E-F) Total bile acid (TBA) concentrations in serum(E) and liver(F). N=6 per group. Error bars are represented by SEM, with individual data points depicted as dots. *p < 0.05, **p < 0.01.


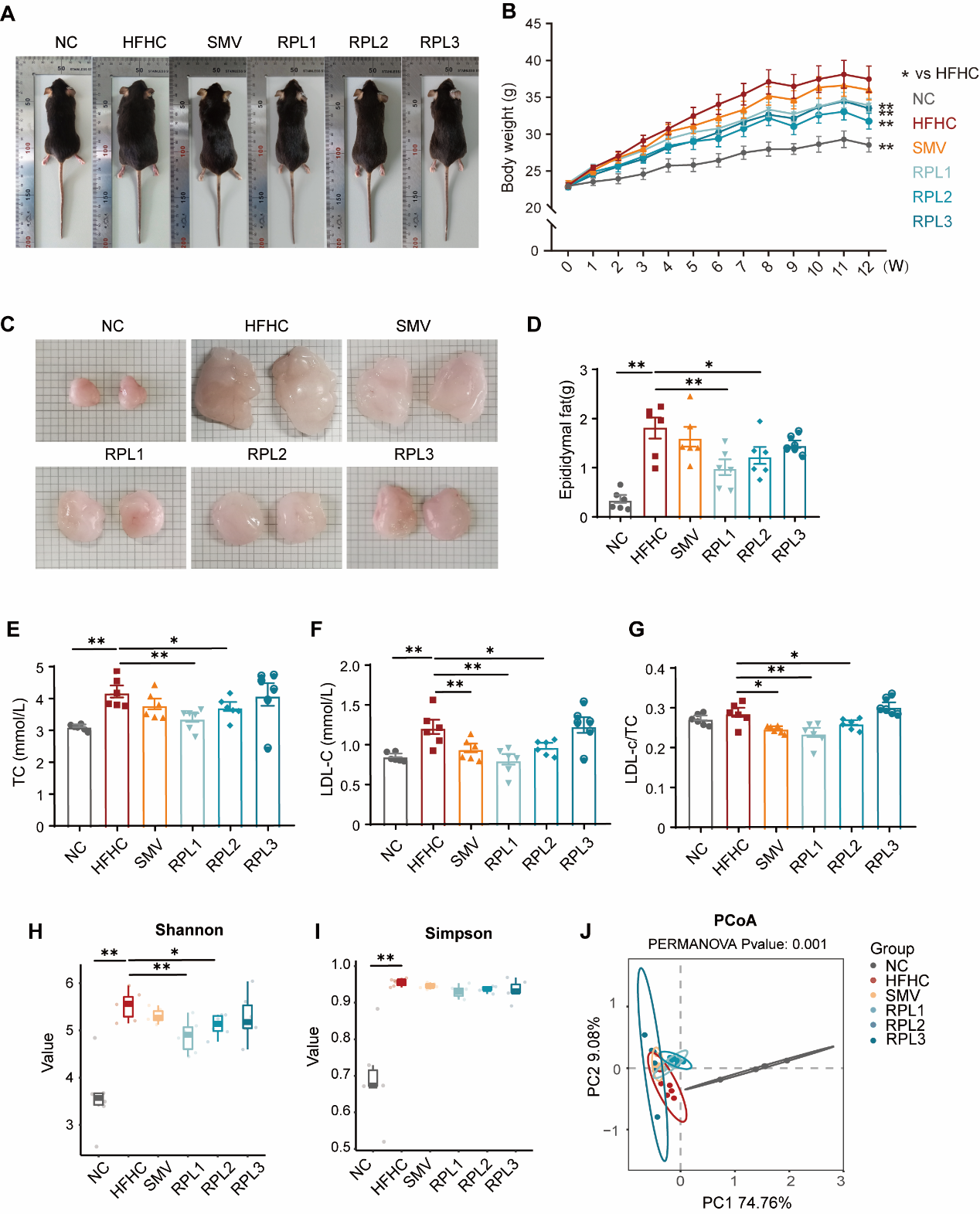


**Fig. S4.** The different effects of gradient doses of the dietary combination on obesity and hyperlipidemia in mice. (A) Representative images of mice at the end of week 12 in the six groups. (B) Changes in body weight of mice in the six groups. (C) Representative images of epididymal fat in the six groups. (D) Changes in epididymal fat weight of mice in the six groups. (E-G) Serum levels of TC(E), LDL-C(F), the ratio of LDL-C to TC(G) at the end of the week 12. (H-I) The α-diversity represented by the Shannon(H) and Simpson(I) indices among the six groups. (J) The β-diversity represented by PCoA based on Bray-Curtis similarity among the six groups. N=6 per group. Error bars are represented by SEM, with individual data points depicted as dots. *p < 0.05, **p < 0.01.

**Supplementary Information for Materials and methods**

**Quantification of Monacolin K (Lovastatin) in Red Yeast Rice**

The red yeast rice was ground, and the lovastatin in it was extracted using 75% ethanol via ultrasonication. The insoluble residue was removed by centrifugation, and the supernatant was subjected to reversed-phase high-performance liquid chromatography (RP-HPLC) to separate the lactone (closed-ring) and acid (open-ring) forms of lovastatin. Detection was performed using a UV detector at a wavelength of 238 nm. A standard curve was established using gradient concentrations of lovastatin standards. The target components were identified by comparing their retention times with those of the standards, while quantification was achieved by comparing the peak area ratios of the target components to those of the standards.

**Western blot**

The liver and small intestine tissues of mice were homogenized in lysis buffer by a tissue homogenizer. Then the mixture was then subjected to centrifugal at 12 000 rpm for 10 min to get supernatant. After mixed with SDS buffer and then heated in a 100 °C metal bath for 10 min, the protein samples were run on SDS-PAGE gels and subsequently transferred to 0.22μm [PVDF](https://www.sciencedirect.com/topics/pharmacology-toxicology-and-pharmaceutical-science/polyvinylidene-fluoride) membranes.

Following the manufacturer's instructions, the membranes were blocked for 30min and incubated with primary antibodies against LDLR (1:500, Abcam, ab52818), [SR-BI](https://www.sciencedirect.com/topics/pharmacology-toxicology-and-pharmaceutical-science/kruppel-like-factor-4) (1:1000, Abcam, ab52629), ABCG5 (1:1000, proteintech,27722-1-AP), ABCG8 (1:1000, Boster, A01482-1), LXRα(1:2000, proteintech,14351-1-AP), IDOL(1:1000, proteintech,15455-1-AP) and β-actin (1:1000, Affinity, AF7018) for approximately 16 h at 4 °C. After that, the membranes were incubated with secondary antibodies (1:3000, Invitrogen) at room temperature for 1 h. The blots were captured by [electrochemiluminescence](https://www.sciencedirect.com/topics/immunology-and-microbiology/electrochemiluminescence) and signals were quantified using Image J.

**RT-qPCR**

Total [RNA](https://www.sciencedirect.com/topics/biochemistry-genetics-and-molecular-biology/rna) was isolated from tissues using commercial RNA extraction kit (Tiangen, China) and reverse-transcribed to complementary double stranded DNA with the Prime Script™ RT reagent kit (Takara, Japan). Then qPCR was conducted using a qPCR SYBR Green Mix Kit (Xinbei, Shanghai, China) for the relative quantification of target mRNA transcripts. β-actin was used as the reference gene. The relative fold of expression change was calculated using 2^−ΔΔCt^ method. The mouse primers were synthesized by Sangon technologies and the primer sequences were listed in the Supplementary Table S2.

**Table S3.** Primer sequences.

| Gene (mouse) | Primer Sequence (5′ to 3′) |
| --- | --- |
| β-actin | GTGACGTTGACATCCGTAAAGA |
|  | GCCGGACTCATCGTACTCC |
| FXR | GCTTGATGTGCTACAAAAGCTG |
|  | CGTGGTGATGGTTGAATGTCC |
| FGF15 | GAAGACGATTGCCATCAAGGA |
|  | CGAATCAGCCCGTATATCTTGC |
| ASBT | CCCAAATGCAACTGTCTGCG |
|  | CACCCCATAGAAAACATCACCA |
| SHP | CAGGTCGTCCGACTATTCTGT |
|  | AGGCTACTGTCTTGGCTAGGA |
| CYP7a1 | GGGATTGCTGTGGTAGTGAGC |
|  | GGTATGGAATCAACCCGTTGTC |
| CYP7b1 | TGGCTTCCTTATCTTGGCATGGC |
|  | TCGCTGATAATCGGCTGCTGAAC |
| CYP27a1 | AGGGCAAGTACCCAATAAGAGA |
|  | TCGTTTAAGGCATCCGTGTAGA |
| CYP8b1 | CCTCTGGACAAGGGTTTTGTG |
|  | GCACCGTGAAGACATCCCC |
| LDLR | CCAATCGACTCACGGGTTCA |
|  | TCACACCAGTTCACCCCTCT |
| SR-BI | TTTGGAGTGGTAGTAAAAAGGGC |
|  | TGACATCAGGGACTCAGAGTAG |
| HMGCR | CTTTCAGAAACGAACTGTAGCTCAC |
|  | CTAGTGGAAGATGAATGGACATGAT |
| ABCG5 | AGGGCCTCACATCAACAGAG |
|  | GCTGACGCTGTAGGACACAT |
| ABCG8 | AGCCTCACTACTCGACGTGAT |
|  | ACTGGGTTGCCCATTTATCCA |
| PCSK9 | TTGCCCCATGTGGAGTACATT |
|  | GGGAGCGGTCTTCCTCTGT |
| SREBP2 | GCAGCAACGGGACCATTCT |
|  | CCCCATGACTAAGTCCTTCAACT |
| LXRα | CTCAATGCCTGATGTTTCTCCT |
|  | TCCAACCCTATCCCTAAAGCAA |
| LXRβ | ATGTCTTCCCCCACAAGTTCT |
|  | GACCACGATGTAGGCAGAGC |
| IDOL | ATGCTGTGCTATGTGACGAGG |
|  | TCGATGATCCCTAGACGCCTG |
| NPC1L1 | CGCCCTTCTTTCTACATGGGT |
|  | GAATCTGCGCTTACGAGGGAG |
| ABCA1 | AAAACCGCAGACATCCTTCAG |
|  | CATACCGAAACTCGTTCACCC |
| ABCG1 | GTGGATGAGGTTGAGACAGACC |
|  | CCTCGGGTACAGAGTAGGAAAG |

**Immunofluorescent staining**

After dewaxing and hydration, tissue sections were performed immunofluorescent staining according to the manufacturer’s instructions of the primary antibody FXR (1:200, Proteintech, 25055-1-AP), ABCG8 (1:50, Boster, A01482-1). Images were taken by a confocal microscopy (Leica, German). The [mean](javascript:;) [fluorescence](javascript:;) [intensity](javascript:;) was analyzed by ImageJ software.

**Gut Microbiota Analysis**

Total genomic DNA was extracted using DNA Extraction Kit (Qiagen) following the manufacturer’s instructions. The extracted DNA was used as template for PCR amplification of bacterial 16S rRNA genes with the barcoded primers and Takara Ex Taq (Takara). Sequencing was performed on an Illumina NovaSeq 6000 with 250 bp paired-end reads by OE Biotech Company (Shanghai, China).

Raw sequencing data were in FASTQ format. Paired-end reads were then preprocessed using Cutadapt software and DADA2 to output the representative reads and the ASV abundance table. All representative reads were annotated and blasted against Silva database (Version 138) using q2-feature-classifier with the default parameters. QIIME2 software was used for alpha and beta diversity analysis. The linear discriminant analysis effect size (LEfSe) method was used to compare the taxonomy abundance spectrum.

**Targeted Metabolomics Profiling**

The targeted metabolomics profiling of feces was performed by APExBIO Technology LLC (Shanghai, China). Analyses were performed using an UHPLC (1290 Infinity LC, Agilent Technologies) coupled to a QTRAP MS (6500+, Sciex). Up to 650 metabolites in 12 biochemical classes were detected.

Briefly, fecal samples were vortexed with cold Methanol/ acetonitrile/water (2:2:1, v/v)，and the supernatant was collected. The sample was then diluted with ice-cold 50% methanol solution and subjected to centrifugation at 4000 × g. The supernatant, mixed with internal standards, was sealed prior for analysis. The analytical parameters were set as follows: HILIC analytical column (2.1mmx100mm, 1.7µm); column temperature at 35℃; mobile phase A（water with 2 mM ammonium formate and 10% acetonitrile）and mobile phase B (acetonitrile with 0.4% formic acid), subsequent C18 analytical columns (2.1x100 mm, 1.7μm); column temperature at 40℃; mobile phase A (water with 5 mM ammonium acetate) and mobile phase B (99.5% acetonitrile). MRM method was used for mass spectrometry quantitative data acquisition. 650 of standard substances was obtained from Sigma-Aldrich (St. Louis, MO, USA). The order of all test samples was randomly selected to reduce the error introduced by the instrument. Quality control (QC) samples were injected every 8 test samples to assess instrument stability and consistency in sample processing.

Metabolites in QCs with coefficient of variation (CV) less than 30 % were denoted as reproducible measurements. A total of 482 fecal cometabolites were quantified in the targeted metabolomics measurements. Any missing values of metabolites included in the analysis were recognized undetected. Analysis was conducted at cloud.metware.cn.
